# Longitudinal Shortening of the Left Ventricle by Cine-CMR for Assessment of Diastolic Function in Patients with Aortic Valve Disease

**DOI:** 10.5935/abc.20190193

**Published:** 2020-02

**Authors:** Sergio Marrone Ribeiro, Clerio Francisco de Azevedo Filho, Roney Sampaio, Flávio Tarasoutchi, Max Grinberg, Roberto Kalil-Filho, Carlos Eduardo Rochitte

**Affiliations:** 1 Universidade Estadual Paulista (UNESP), Botucatu, SP - Brazil; 2 Universidade do Estado do Rio de Janeiro (UERJ), Rio de Janeiro, RJ - Brazil; 3 Instituto do Coração (InCor) - Universidade de São Paulo (USP), São Paulo, SP - Brazil

**Keywords:** Cardiovascular Diseases/mortality, Cardiomyopathy, Hypertrophic/complications, Diagnostic Imaging, Echocardiography, Magnetic Resonance Spectroscopy, Heart Failure, Aortic Valve Insufficiency

## Abstract

**Background:**

Diastolic dysfunction, commonly evaluated by echocardiography, is an important early finding in many cardiomyopathies. Cardiac magnetic resonance (CMR) often requires specialized sequences that extends the test time. Recently, feature-tracking imaging has been made available, but still requires expensive software and lacks clinical validation.

**Objective:**

To assess diastolic function in patients with aortic valve disease (AVD) and compare it with normal controls by evaluating left ventricular (LV) longitudinal displacement by CMR.

**Methods:**

We compared 26 AVD patients with 19 normal controls. Diastolic function was evaluated as LV longitudinal displacement in 4-chamber view cine-CMR images using steady state free precession (SSFP) sequence during the entire cardiac cycle with temporal resolution < 50 ms. The resulting plot of atrioventricular junction (AVJ) position versus time generated variables of AVJ motion. Significance level of p < 0.05 was used.

**Results:**

Maximum longitudinal displacement (0.12 vs. 0.17 cm), maximum velocity during early diastole (MVED, 0.6 vs. 1.4s^-1^), slope of the best-fit line of displacement in diastasis (VDS, 0.22 vs. 0.03s^-1^), and VDS/MVED ratio (0.35 vs. 0.02) were significantly reduced in AVD patients compared with controls, respectively. Aortic regurgitation showed significantly worse longitudinal LV shortening compared with aortic stenosis. Higher LV mass indicated worse diastolic dysfunction.

**Conclusions:**

A simple linear measurement detected significant differences on LV diastolic function between AVD patients and controls. LV mass was the only independent predictor of diastolic dysfunction in these patients. This method can help in the evaluation of diastolic dysfunction, improving cardiomyopathy detection by CMR, without prolonging exam time or depending on expensive software.

## Introduction

Diastolic dysfunction is an early marker of cardiac disease and precedes systolic dysfunction. It can occur in the presence or absence of symptoms and with normal or abnormal systolic function.^1,2^ There is a high morbidity and mortality associated with this condition due to the potential transition to diastolic heart failure, but it may be underdiagnosed because of the diagnostic criteria. Diastolic dysfunction has an increasing incidence with age and is associated with diabetes mellitus, atrial fibrillation, coronary artery disease, pulmonary hypertension,^3-6^ and congenital heart diseases. Left ventricular (LV) hypertrophy has been associated with impaired diastolic function, which is commonly described in systemic hypertension, aortic valve diseases and hypertrophic cardiomyopathy.^7-9^

Echocardiography is the most used technique for diastolic dysfunction evaluation in daily clinical routine. Cardiovascular magnetic resonance (CMR) has been widely used for the evaluation of LV morphology and systolic function due to its excellent image quality and lack of geometric assumptions.^9^ However, CMR is less used for evaluating diastolic function despite the development of several relevant techniques,^10^ including the use of volumetric filling curves,^11^ phase-contrast imaging,^12^ myocardial tissue tagging,^13^ and strain-encoded imaging.^14^ The reasons for the limited utilization of these techniques in clinical practice are the time-consuming processes for additional image acquisition and post-processing. For instance, obtaining LV volume curves over the entire cardiac cycle, with the mandatory tracking of endocardial and epicardial contours for all cardiac phases in a cine-CMR series takes a long time and requires a specialized software with automated contour detection. Additionally, other specialized techniques of diastolic dysfunction evaluation require additional sequences of images, such as phase-contrast images, which mean longer CMR exam time. In two recent publications by Saba et al.^9^ and Dusch et al.,^7^ CMR longitudinal LV shortening has been shown to be useful for diastolic dysfunction assessment.

In the current study, we hypothesized that patients with severe aortic valve stenosis or regurgitation and preserved ejection fraction have diastolic LV dysfunction defined by motion of the atrioventricular junction (AVJ) at CMR.

## Methods

### Study population

We retrospectively identified 26 patients with severe aortic valve disease (AVD) and normal ejection fraction, who underwent CMR and were scheduled for aortic valve replacement surgery, and 19 normal control subjects. Eleven of AVD patients (42.3%) had predominantly aortic insufficiency, and 15 of them (57.7%) had predominantly aortic stenosis. This sample size was based on the number of patients with confirmed diagnosis, available for analysis.

The patients were clinically followed up at valve disease outpatient clinic of our institution. The exclusion criteria were: age under 18 and over 85 years old, diabetes mellitus, systemic arterial hypertension, dyslipidemia or concomitant significant coronary artery disease. All patients over 40 years old had a coronary angiography, and those with significant coronary artery disease (luminal stenosis >50%) were excluded. Patients with concomitant mitral valve disease were also excluded, as well as the ones with previous cardiac surgery and contraindications for CMR such as pacemaker use, metal clips or other ferromagnetic structures and claustrophobia.

Healthy volunteers with no significant past medical history had been recruited to establish baseline AVJ motion values. In addition, 19 healthy volunteers (10 men), aged between 24 and 58 years old, without hypertension, diabetes mellitus, coronary artery disease or other significant past medical history, and all with normal CMR examinations were used for comparison with the 26 AVD patients.

The CMR tests were performed with a 1.5 Tesla clinical scanner (Signa CV/i, GE Medical Systems, Waukesha, Wisconsin/USA) and dedicated cardiac surface phased-array coil. After localization of the heart, eight to 12 contiguous short-axis slices (8.0 mm slice thickness and 2mm gap between the slices), encompassing the entire LV and 4 long-axis slices were selected. The analysis was performed in a four-chamber view. Cine images were acquired with a steady-state free precession pulse sequence (SSFP) with temporal resolution of less than 50 ms and standard parameters: TR 3.9 ms, TE 1.8 ms, flip angle 45°, receiver bandwidth ± 125 kHz, field of view (FOV) of 34 x 34 cm, 256x160 matrix, voxel size 1.3 x 2.1 x 8.0mm.

### Image and data analyses

**Figure 1:**
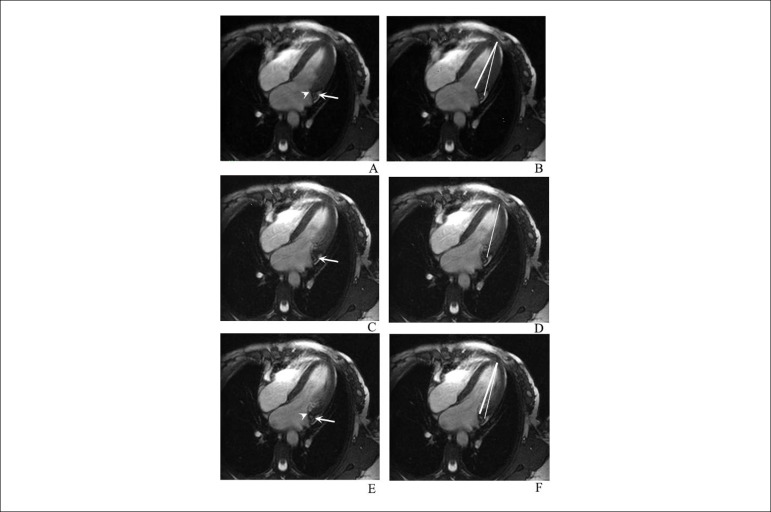
Longitudinal displacement of the atrioventricular junction (AVJ). The same four- chamber slice is shown in three different cardiac phases during AVJ rapid movement: A and B, C and D, E and F. On the left column the arrowhead represents the reference used by Saba et al.^9^ and the thin arrow shows the anatomical reference used in the present study. Note that when the cardiac motion is faster (small arrow in C and D), we could not precisely identify the site of the mitral valve insertion; however, the adjacent coronary sinus wall is still well defined. On the right column, we showed the lines used for the LV longitudinal measurements on this study (thinner line) and by Saba et al.^9^ (thicker line)

The longitudinal motion of the AVJ was tracked through the cardiac cycle over 20 cardiac phases, on four-chamber view SSFP cine CMR images. The baseline position of the AVJ was defined at end diastole and its longitudinal displacement was measured relative to a reference line drawn between the LV apex (epicardial border, hypointense line corresponding to interface of myocardium and epicardial fat) and the inferior limit (hypointense line) of the coronary sinus running through the AV groove, immediately lateral to AVJ. These specific landmarks showed clear visualization on the cine-MR SSFP images and allowed for a robust tracking throughout the cardiac phases, with minimal blurring or loss of image definition. We did not use the midpoint of the mitral annulus^9^ as we aimed to find the septal and lateral AVJ precisely; we also simplified the measure when we traced a unique line with well-defined landmarks. A simple straight line was traced between basal and apical landmarks using the Webpax software tool (Heart Imaging Technologies, LLC. Durham, NC, USA) (Figure 1). This line is a regular caliper available in all softwares capable of visualization of DICOM images.

**Figure 2:**
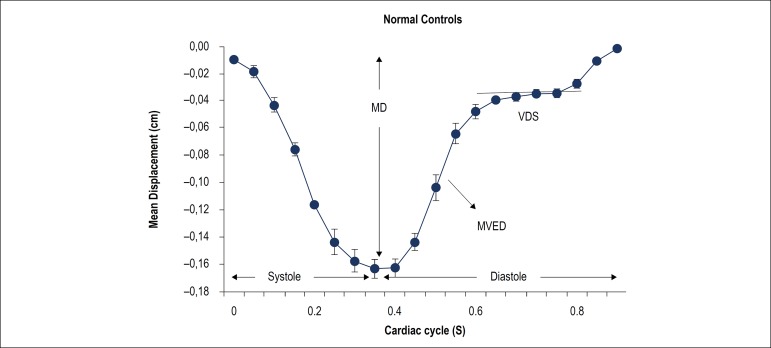
Atrioventricular junction (AVJ) displacement-versus-time plot of the normal controls. AVJ position at multiple time points during the cardiac cycle. Error bars represent one standard deviation above and below the mean. MD: maximum displacement; MVED: maximum velocity early diastole; VDS: velocity in diastasis.

LV longitudinal lengths were divided by the longitudinal length at end diastole (maximum length) to provide a percent reduction of longitudinal length, corrected for individual heart sizes. Based on the plots of AVJ position versus time in the cardiac cycle (Figure 2), four motion variables were calculated: maximum longitudinal displacement (MD) of the AVJ, maximum velocity during early diastole (MVED), slope of the best-fit line of AVJ velocity in diastasis (VDS), and the ratio of VDS/MVED. The MVED values for each patient were calculated according to the time-versus-displacement graph, a linear regression (straight line) was adjusted for early diastole (slope). The same method was used for VDS considering now the diastasis time. All measurements were performed by two independent blinded radiologists. Cine-CMR images were used for the assessment of LV volume, mass, and function.

### Statistical analysis

Continuous variables of the AVJ motion are presented as means and standard deviation. Normality distribution was assessed by Shapiro-Wilk test. Data obtained in AVD patients were compared to normal control subjects using the unpaired Student t-test. The categorical variables are presented in percentage.

Bland-Altman plots were used to compare LV displacement parameters between patients and controls obtained by two independent blinded observers.

The SAS System and SPSS statistical software packages were used for data analysis with a significance level of p < 0.05.

## Results

Patients’ age ranged between 26 and 72 years old, with 19 men and seven women. The mean age of AVD patients and healthy volunteers in the study was 46.8 ± 13.7 and 43.1± 11.8 years, respectively. All patients were symptomatic, complaining of exertional dyspnea, angina, and syncope (Table 1). Indexes of LV volume and mass are shown in Table 2. All patients had normal or mild reduction of ejection fraction reduction (mean LV ejection fraction of 53.1 ± 9.9%). As expected, patients with predominant aortic regurgitation showed an eccentric hypertrophy pattern with end-diastolic volume and end-systolic volume significantly increased when compared with patients with predominant aortic stenosis, who presented a concentric hypertrophy pattern.

**Table 1:** Characteristics of patients with aortic valve disease and controls

	Aortic regurgitation	Aortic stenosis	Controls	p
n (%)	11(42.3)	15 (57.7)	19	
Age, years	46.0 ± 15.7	48.7 ± 11.3	38.1 ± 10.5	0.610/0.039*
Men, n (%)	10(90.9)	9(60.0)	10(52.6)	0.079/0.101*
Weight (kg)	76.6 ± 10.6	71.2 ± 11.9	67.9 ± 15.3	0.336/0.356*
BMI (kg/m^2^)	27.9 ± 3.5	26.3 ± 3.8	23.5 ± 3.6	0.382/0.021*
**Etiology**				
Rheumatic	9(81.8)	3(20.0)	-	
Bicuspid	2(18.2)	8(53.3)	-	
Degenerative/Calcification	0 (0.0)	4(26.7)	-	0.007
**NYHA Functional class**				
I	1(9.1)	0(0.0)	19(100.0)	
II	7(63.6)	8(53.3)	0(0.0)	
III	3(27.3)	7(46.7)	0(0.0)	0.526
Heart rate, bpm	65.0 ± 11.9	81.5 ± 20.7	70.1 ± 10.6	0.027/0.019*
SBP	126.7 ± 15.1	121.5 ± 15.2	111.6 ± 8.98	0.505/0.018*
DBP	80 ± 8.9	71.8 ± 12.8	71.3 ± 6.6	0.183 / 0.143*
Angina	0(0.0)	1(6.7)	-	0.465
Syncope	0(0.0)	1(6.7)	-	0.465
Hypertension	6(54.6)	6(40.0)	-	0.100
Diabetes	0(0.0)	1(13.3)	-	0.342
Hypercholesterolemia	0(0.0)	0(0.0)	-	-
Smoking	0(0.0)	5(33.3)	-	0.100
Family History of CAD	4(36.4)	3(20.0)	-	0.190

BMI: body mass index; NYHA: New York Heart Association; SBP: systolic blood pressure; DBP: diastolic blood pressure; CAD: coronary artery disease;

* comparison the three groups including controls; remaining p values for comparison between aortic regurgitation and stenosis only.

**Table 2:** Cardiac magnetic resonance parameters of patients with aortic valve disease and controls

	Aortic regurgitation	Aortic stenosis	Controls	p
n (%)	11(42.3)	15 (57.7)	19	
LVEDV, ml	299.6 ± 68.5	179.99 ± 42.1	129 ± 24.7	< 0.001
LVESV, ml	148.9 ± 60.4	82.0 ± 28.7	45.5 ± 9.4	< 0.001
LVEF, %	51.7 ± 11.4	55.1 ± 9.1	64.7 ± 5.3	< 0.001
LV mass, g	264.2 ± 42.4	272.8 ± 45.5	118.1 ± 40.5	< 0.001
Eccentric Hypertrophy, n (%)	10(90.9)	1(6.7)	-	
Concentric Hypertrophy, n (%)	1(9.1)	14(93.3)	-	< 0.001

LVEDV: left ventricular end-diastolic volume; LVESV: left ventricular end-systolic volume; LVEF: left ventricular ejection fraction; LV: left ventricular. Definition criteria of concentric hypertrophy is LV mass to LVEDV ratio > 1.16 g/ml.

### AVJ motion analysis

**Figure 3:**
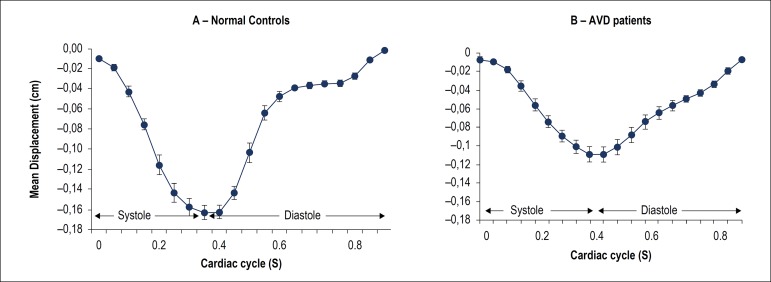
Displacement-versus-time plot in normal controls (A) and aortic valve disease patients (B). Error bars represent one standard deviation above and below the mean. AVD: aortic valve disease.

**Figure 4:**
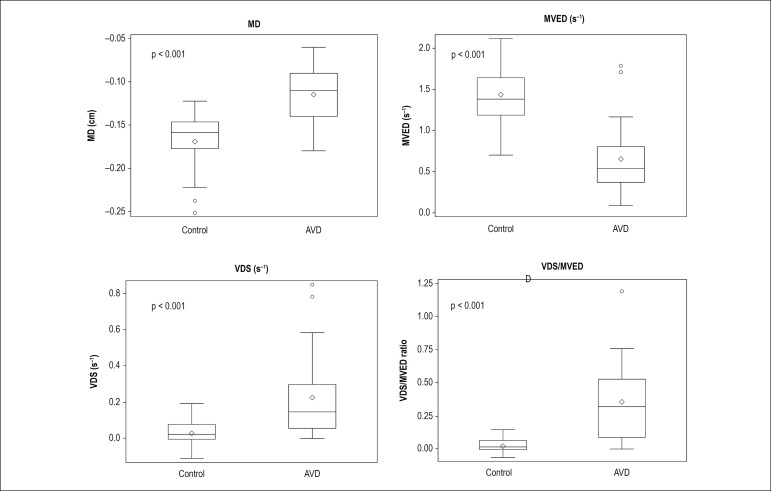
Box plots of the variables of atrioventricular junction motion in patients with aortic valve disease and healthy volunteers. In both groups, the box plots display the sample minimum (lower whisker), lower quartile (lower box subdivision), median (horizontal band), upper quartile (upper box subdivision), and sample maximum (upper whisker) for each of the AVJ motion variables - maximum displacement (MD); maximum velocity early diastole (MVED); velocity diastasis (VDS) and VDS/MVED. Circles indicate outliers (p < 0.0001 for all)

Means and standard deviations were calculated for each of the AVJ motion variables (MD, MVED, VDS, VDS/MVED) of AVD patients and normal control subjects. AVJ data were compared between patients and controls. We found statistically significant differences in MD and the three CMR correlates of diastolic LV function (MVED, VDS, VDS/MVED) in patients with AVD compared to normal controls, as noted in Table 3 and Figure 3. Patients with AVD showed significantly lower normalized MD at the AVJ compared to healthy volunteers. AVJ of patients with AVD recoiled at significantly slower normalized maximum velocities (s^-1^) in early diastole compared with healthy volunteers. Conversely, during diastasis, AVJ motion occurred at significantly faster normalized velocities in patients with AVD. We found a 17-fold higher VDS/MVED ratio in AVD compared with healthy volunteers (Figures 3 and 4, Table 3).

**Table 3:** Comparison of atrioventricular motion variables between patients with aortic valve disease (AVD) and healthy volunteers

	Control	AVD	p
MD (cm)	-0.169 ± 0.034	-0.115 ± 0.035	< 0.0001
MVED (s^-1^)	1.439 ± 0.388	0.65 ± 0.413	< 0.0001
VDS (s^-1^)	0.029 ± 0.069	0.224 ± 0.232	< 0.0001
VDS/MVED	0.021 ± 0.051	0.352 ± 0.292	< 0.0001

MD: maximum displacement; MVED: maximum velocity early diastole; VDS: velocity in diastasis.

**Figure 5:**
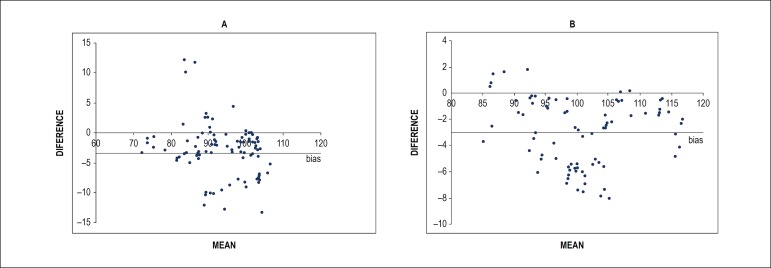
Interobserver comparison of maximum displacement measures in normal controls (A) and patients with aortic valve disease (AVD) (B).

The Bland-Altman analysis (Figure 5) for MD revealed a bias of -2.81 and 95% CI (confidence interval) of (-3.66 to -1.95) for normal controls (p < 0.001) and a bias of -2.97, 95% CI of (-4.11 to -1.83) for AVD patients with p < 0.001.

Comparison of diastolic function based on AVJ parameters between patients with predominant stenosis and predominant regurgitation did show significant differences in all diastolic function at CMR (Table 4). Impairment of diastolic function was higher in patients with aortic regurgitation compared to stenosis.

**Table 4:** Atrioventriculr junction motion variables of patients with predominant aortic stenosis and aortic regurgitation

	Stenosis	Regurgitation	p
MD (cm)	-0.130 ± 0.036	-0.093 ± 0.018	0.0026
MVED(s^-1^)	0,790 ± 0.479	0.470 ± 0.200	0.0312
VDS(s^-1^)	0.317± 0.262	0.097 ± 0.093	0.0075
VDS/MVED	0.440 ± 0.295	0.231 ± 0.252	0.0703

AVJ: atrioventricular junction; MD: maximum displacement; MVED: maximum velocity early diastole; VDS: velocity diastasis.

### Diastolic function, LV structure and clinical parameters

Results of univariate and multiple linear regression analysis including LV mass, volumes and function as well as patient characteristics such as age, gender, heart rate and blood pressure are shown in Table 5.

**Table 5:** Univariate and multiple linear regression analysis (p-values) for the prediction of the diastolic function parameters derived from linear measurements

	MD		MVED		VDS		VDS/MVED	
	Univariate	Multiple Linear Regression	Univariate	Multiple Linear Regression	Univariate	Multiple Linear Regression	Univariate	Multiple Linear Regression
Age	0.087		0.059		0.912		0.130	
Gender	0.070		0.272		0.819	0.04	0.705	
LV mass	< 0.001	< 0.001	< 0.001	0.001	0.001	< 0.001	0.002	0.003
LVEDV	< 0.001		< 0.001		0.366		0.154	
LVESV	< 0.001		< 0.001		0.605		0.083	
LVEF	0.002		< 0.001		0.610		0.004	0.006
HR	0.886		0.645		< 0.001	0.081	0.025	
SBP	0.140		0.028		0.399		0.051	
DBP	0.190		0.616		0.846		0.232	

MD: maximum displacement; MVED: maximum velocity early diastole; VDS: velocity diastasis; LV: left ventricle; LVEDV: end-diastolic volume; LVESV: end-systolic volume; LVEF: left ventricular ejection fraction; HR: heart rate; SBP: systolic blood pressure; DBP: diastolic blood pressure.

In a univariate analysis, MD and MVED correlated significantly with LV volume, left ventricular ejection fraction (LVEF) and LV mass. MVED also correlated to systolic blood pressure (SBP). VDS/MVED correlated with LV mass, LVEF and heart rate (HR). VDS showed correlation only with LV mass and HR (Table 5). In a multivariate linear regression model, MD and MVED were predicted only by LV mass. All other parameters of LV structure, volume and function were not predictive of MD and MVED in this multivariate approach. Gender and LV mass independently predicted VDS, while VDS/VMED ratio was predicted by LV mass and LVEF.

In summary, these results indicate that LV mass maintains a significant correlation across the four measured diastolic parameters in the univariate and forward stepwise multiple linear regression. Additionally, gender maintained a significant correlation with VDS and LVEF with VDS/MVED ratio. The remaining variables were not independently correlated with diastolic function parameters derived from linear measurements. Thus, body size (body mass index), HR and blood pressure did not influence significantly linear diastolic parameters measured by CMR.

## Discussion

Novel correlates of diastolic LV function measured by CMR originally investigated in this study were markedly abnormal in patients with AVD. Measured at the AVJ, patients with AVD had significantly lower maximum displacement, slower velocity during early diastolic filling, and higher velocity during diastasis compared to normal control subjects.

Saba et al.^9^ reported diastolic LV function alterations evaluated through the AVJ motion by CMR in patients with hypertrophic cardiomyopathy compared to normal control patients. Results from our control group were very similar to those reported by these authors, although with slightly greater values mainly because we used a more lateral anatomical landmark. Also, we used only one measurement instead of two of AVJ displacement, hence adopting one well-defined reference point of the AVJ lateral wall, in a more simplified method.

### LV hypertrophy and diastolic function

LV hypertrophy is a recognized risk factor for cardiac morbidity and mortality^15^ and is associated with systolic and/or diastolic function disturbances.^16-18^ In patients with AVD, diastolic and systolic function disturbances have important implications for morbidity and mortality, before and after aortic valve replacement.^16-21^ In the study by Lamb et al.,^22^ the ejection fraction was largely unaffected in the group of patients with severe AVD, suggesting that a deterioration of the ejection fraction should be considered as a sign of severe and advanced disease,^22^ which was corroborated by other authors.^20,22^ After aortic valve replacement, LV diastolic function improves, as indicated by parameters of transmitral flow.^22^ In our results, we not only detect diastolic dysfunction in AVD patients compared to normal controls, but also demonstrated a worse diastolic dysfunction in patients with aortic regurgitation. LV mass was significantly and independently correlated with all linear measurements of diastolic function evaluated by CMR.

### Diastolic dysfunction evaluation

Phase contrast magnetic resonance imaging allows measurement of flow velocity as well as flow volumes across the mitral valve orifice, providing a new means of diastolic function assessment that may be even more sensitive than Doppler echocardiography. Although it is a well-established tool to assess systolic dysfunction, it is rarely used clinically to assess LV diastolic function, which may require additional dedicated sequences and extensive post-processing.^7^ In this sense, in a recently published study by Dusch at al.,^7^ similar to our study, the authors used a horizontal-long axis SSFP sequence, which they called midwall longitudinal fractional shortening. They verified the percentage of shortening of the distance from the anterior leaflet mitral valve basis to apical endocardium in diastole in relation to systole, comparing these measures to the echocardiogram of 80 patients with varied cardiomyopathies and different degrees of diastolic function.^23,24^ Using a simpler method than the one used in the present study, Dusch at al.^7^ were able to detect that the midwall longitudinal fractional shortening of grade II/III was significantly lower than that of grade 0/I.

Our study shows many advantages of using this new and accurate method for evaluation of LV diastolic function. It does not require the development of an acquisition sequence or post-processing software, and LV diastolic function can be easily evaluated by existing equipment. LV diastolic function can be retrospectively evaluated if prior cine image datasets were stored.

Nonetheless, our study has several limitations. One of the most significant ones in terms of practicality is the need to perform manually 20 linear measurements in each phase of one cardiac cycle. However, the use of more automated software would help in a faster measurement. Another significant limitation was that AVJ motion and echocardiography variables were not directly correlated. It is possible that LV longitudinal displacement and the velocity of this displacement might suffer influence from LV geometric morphology. However, the LV dimensions measured as LV end-diastolic volume and diameter did not have significant effect on diastolic parameters. Additionally, LV longitudinal displacements are, by definition, normalized by the LV longitudinal dimension, and the other dimensions are incorporated to volume measurements. Despite the practical advantages of using this new method for LV diastolic function evaluation, it would be important, in future studies, to evaluate its accuracy compared to other existing methods in general evaluation of cardiac diseases that cause LV diastolic dysfunction, verifying the sensitivity and specificity in classifying different diastolic dysfunction degrees. Another limitation of the present study was the relatively small number of AVD patients.

Finally, we have demonstrated that diastolic function evaluation can be performed by the SSFP cine sequences routinely acquired by conventional CMR tests, with no need for additional specific sequences or specific software. The incorporation of this technique to clinical routine would improve the CMR ability to analyze diastolic function, even retrospectively using previously acquired CMR images.

## Conclusion

In conclusion, LV longitudinal shortening is a quick and reliable technique for assessment of diastolic dysfunction in AVD patients that can be performed in routine CMR studies without the use of specific or sophisticated software.

The AJV curve showed significant differences in all diastolic parameters analyzed between AVD patients and normal controls. Further studies should confirm that this method is valuable for other cardiac diseases.
